# Disruption of the folate pathway in zebrafish causes developmental defects

**DOI:** 10.1186/1471-213X-12-12

**Published:** 2012-04-05

**Authors:** Marina S Lee, Jenna R Bonner, David J Bernard, Erica L Sanchez, Eric T Sause, R Reid Prentice, Shawn M Burgess, Lawrence C Brody

**Affiliations:** 1 National Human Genome Research Institute, National Institutes of Health, Bethesda, MD, USA; 2 Present address: University of Maryland School of Medicine, Baltimore, MD, USA; 3 Present address: University of Washington, Seattle, WA, USA; 4 Present address: National Cancer Institute, Frederick, MD, USA; 5 Present address: Illumina Inc, San Diego, CA, USA

## Abstract

**Background:**

Folic acid supplementation reduces the risk of neural tube defects and congenital heart defects. The biological mechanisms through which folate prevents birth defects are not well understood. We explore the use of zebrafish as a model system to investigate the role of folate metabolism during development.

**Results:**

We first identified zebrafish orthologs of 12 human folate metabolic genes. RT-PCR and in situ analysis indicated maternal transcripts supply the embryo with mRNA so that the embryo has an intact folate pathway. To perturb folate metabolism we exposed zebrafish embryos to methotrexate (MTX), a potent inhibitor of dihydrofolate reductase (Dhfr) an essential enzyme in the folate metabolic pathway. Embryos exposed to high doses of MTX exhibited developmental arrest prior to early segmentation. Lower doses of MTX resulted in embryos with a shortened anterior-posterior axis and cardiac defects: linear heart tubes or incomplete cardiac looping. Inhibition of *dhfr* mRNA with antisense morpholino oligonucleotides resulted in embryonic lethality. One function of the folate pathway is to provide essential one-carbon units for dTMP synthesis, a rate-limiting step of DNA synthesis. After 24 hours of exposure to high levels of MTX, mutant embryos continue to incorporate the thymidine analog BrdU. However, additional experiments indicate that these embryos have fewer mitotic cells, as assayed with phospho-histone H3 antibodies, and that treated embryos have perturbed cell cycles.

**Conclusions:**

Our studies demonstrate that human and zebrafish utilize similar one-carbon pathways. Our data indicate that folate metabolism is essential for early zebrafish development. Zebrafish studies of the folate pathway and its deficiencies could provide insight into the underlying etiology of human birth defects and the natural role of folate in development.

## Background

Folate one-carbon metabolism (FOCM) plays an integral role in human development and disease. Genetic variants in folate pathway genes are associated with increased risk for neural tube defects (NTD) (and other congenital defects) [1], cardiovascular disease [2], cancer [3] and cognitive decline [4]. Women taking supplemental folic acid prior to conception significantly reduce their chance of having an NTD affected pregnancy [5]. The mechanism by which folic acid reduces the incidence of NTD’s has not been fully elucidated.

**Figure 1:**
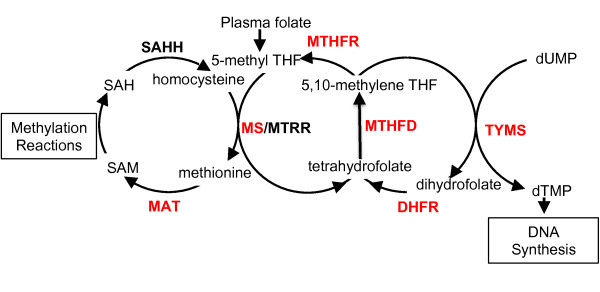
**Folate mediated one-carbon cycle.** Folic acid (vitamin B9) is a cofactor in one-carbon metabolism, acting as a shuttle for methyl groups that will be used in the metabolism of s-adenosyl methionine (SAM), *de novo* synthesis of purines and thymidylate. Dihydrofolate reductase (dhfr), an enzyme involved in the recycling of this cofactor, reduces dihydrofolate to tetrahydrofolate (THF), thereby preparing the folate derivative for subsequent methyl group addition. This enzyme is competitively inhibited by methotrexate (MTX), a chemotherapeutic drug with a thousand fold higher binding affinity for *dhfr* then its natural substrate, dihydrofolate TYMS – thymidylate synthetase, MTHFD – methylenetetrahydrofolate dehydrogenase, MTHFR – methylenetetrahydrofolate reductase, MS – methionine synthase, MTRR – methionine synthase reductase, MAT- Methionine adenosyltransferase, SAHH - s-adenosyl homocysteine hydrolase, SAH – s-adenosyl homocysteine. Enzymes in red are discussed in this paper.

Neural tube defects are a common congenital malformation affecting 1 in 1000 conceptuses. In humans, neural tube closure occurs by day 28 post-conception and involves a complex co-ordination of morphogenic activities and regulatory processes. Events this early in development are not amenable to study in humans and difficult to observe in mammalian model organisms. The mouse is a well-developed model organism for studying neural tube defects and the role of folate, however the research is limited by litter size and stage at which the defects can be studied [6]. For example, homozygous mutants in methionine synthase, an enzyme in the folate pathway (Figure 1), are embryonic lethal and are resorbed prior to E9.5 [7]. While the role of the folate pathway during development is being investigated in the invertebrate model organisms *Drosophila*[8] and *C. elegans*[9] , these models are evolutionarily distant from vertebrates. As with the invertebrate models, zebrafish can be studied in large numbers and are more closely related to mammals. The mechanical and structural aspects of neural tube formation in zebrafish differ from that found in mammals [10] but the genetic programs of notochord formation and development are shared between fish and humans [11]. The significant advantages of working with zebrafish (external fertilization, large clutch size, and transparency) may render it a useful investigative model for studying the role of folate in development and disease.

Folate, also known as vitamin B_9_, is a cofactor for enzymes involved in cellular transmethylation reactions and *de novo* synthesis of purines and thymidylate [12]. In its reduced form, the folate molecule acts a carrier of single carbon units. The ultimate donors of the methyl groups are serine, glycine, histidine and formate. Methylated folates are cofactors in two steps during inosine synthesis (the precursor of purine bases) and in the synthesis of thymidylate (a precursor necessary for DNA synthesis). The third major cellular reaction using folates as a co-factor is the methionine cycle, whereby the methyl group is added to homocysteine to form methionine. The subsequent product s-adenosyl-methionine is used in the synthesis of neurotransmitters, methylation of phospholipids, DNA methylation, histone methylation and mRNA capping. Folate dependent enzymes are found in the mitochondria, cytoplasm, and nucleus. Folate is a water-soluble vitamin found primarily in leafy vegetables. Due to its protective effect in early pregnancy, folate fortification is mandated in the US with synthetic folate in the form of folic acid added to milled grain products.

We sought to evaluate the zebrafish as a model for studying vertebrate folate and one-carbon metabolism. We aimed to understand the role of FOCM in early organ development. There is little to no information on these pathways in fish. Using bioinformatics we have identified zebrafish orthologs of 12 human folate metabolic genes and characterized their spatial and temporal expression patterns. Chemical and genetic disruption of these pathways demonstrated that the folate pathway is essential for several aspects of zebrafish development, as treated embryos are embryonic lethal. We show cell cycle perturbations as a result of defects in FOCM. We hypothesize these defects are the result of reduced folate substrates for thymidylate synthase, a rate-limiting enzyme in DNA synthesis. Zebrafish studies of the folate pathway and its deficiencies could provide insight into the underlying etiology of human birth defects and the natural role of folate in development.

## Results

### Cloning & RT-PCR

**Figure 2:**
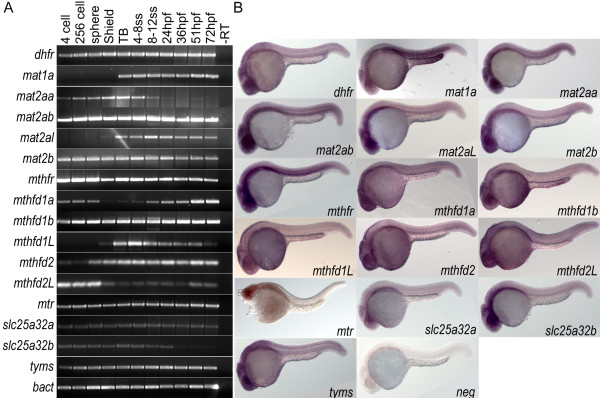
**Wild type expression data for folate pathway transcripts.** A) RT-PCR analysis of 11 embryonic stages within the first 72 hours of development indicates that genes involved in the folate pathway are generally maternally loaded transcripts that are expressed throughout early development. B) *in situ* hybridization for these 16 genes at 24 hpf shows that many are globally expressed with enrichment in anterior neural tissues including tectonic and retinal staining. Specific somatic staining is seen in *mat1a, mat2aa, mat2ab,* and *mthfr*. YSL staining is also observed in *mat1a* and *mthfd1b.* Embryos in lateral view.

Orthologs of eleven enzymes and one transporter involved in folate-one carbon metabolism were identified in zebrafish by BLAST searches, cloned, and characterized (Figure 1 and Table 1). The six previously un-annotated genes were sequenced and submitted to the ZFIN nomenclature committee (*mat2aa*, *mat2ab*, *mat2al*, *mthfr*, *mthfd1a*, and *mthfd1b*) and updated sequences were submitted to GenBank. Expression of these genes during development was measured via RT-PCR and RNA *in situ* hybridization. Most genes identified in this pathway appear to be ‘house keeping’ genes that are maternally loaded, present throughout early embryonic development, and whose expression is predominantly enriched in anterior CNS (Figure 2).

**Table 1:** Folate Pathway Protein Homologies

**zfin**	**Human Protein**	**% Identity**	**% Similarity**
mat1a	NP_000420.1	84	94
mat2aa* ^#^	NP_005902.1	91	96
mat2ab* ^#^	"	89	96
mat2al*	"	mat1a 75/mat2a 73	nd
mat2b	NP_037415.1	65	79
tyms	NP_001062.1	82	91
dhfr	NP_000782.1	61	76
mthfr* ^#^	NP_005948.3	79	89
mthfd1a*	NP_005947.3	57	74
mthfd1b*	"	77	88
mthfd1l	NP_056255.2	80	89
mthfd2	NP_006627.2	80	91
mthfd2L	NP_001138450.1	67	82
mtr	NP_000245.2	78	89
slc25a32a	NP_110407.2	66	81
slc25a32b	"	70	82

nd – Not done.

* Gene name assigned as a result of this work.

^#^ Updated sequence submitted to GenBank.

Dihydrofolate reductase (Dhfr), a key enzyme in the conversion of dietary folate into the reduced bioactive form that can be utilized by cells (EC 1.5.1.3), is found in the zebrafish genome on chromosome 5. The predicted protein has 61% identity (76% similarity) to the human protein (NP_000782.1) [13]. This transcript is maternally loaded and is present throughout the first 72 hours of development. At 24hpf the transcript is enriched in neural tissues; alar plate midbrain region, CNS, optic tectum, and optic vessels ([14], Figure 2B).

Methionine adenosyltransferases (MAT) are a family of enzymes that catalyze the synthesis of s-adenosylmethionine from methionine and ATP (EC 2.5.1.6). Folate is a cofactor in the preceding reaction that generates the methionine from homocysteine. *Mat1a* encodes the liver specific forms of this enzyme, MATI and MATIII [15]. *Mat2a* is expressed throughout the organism and encodes MATII. The regulatory subunit for MATII is encoded by *mat2b*[16]. Orthologs of these genes are found in zebrafish. *Mat1a* is located on chromosome 13 and has 84% identity (94% similarity) to the human protein (NP_000420.1). The transcript for this gene is not expressed until after the onset of gastrulation, by 24 hpf its expression is enriched in the YSL and myotomes ([14], Figure 2). *Mat2b* is found on chromosome 21 and has 65% identity (79% similarity) to the human protein (NP_037415.1). This is a maternally loaded transcript and is expressed throughout early zebrafish development. At 24 hpf, its expression is enriched in anterior neural tissues (Figure 2). The zebrafish genome has two paralogs of human Mat2a (NP_005902.1): Mat2aa, with 91% identity (96% similarity) on chromosome 10 and Mat2ab with 89% identity (96% similarity) on chromosome 5. *Mat2aa* is a maternally expressed gene whose expression decreases as development progresses. *Mat2ab* is a maternally loaded transcript and is present throughout the early stages of development. Both these transcripts are enriched in myotomes and anterior neural structures at 24 hpf ([14], Figure 2). The zebrafish genome contains another paralog in the *mat* family, which we propose to name Mat2a-like (mat2al), located on chromosome 10. This gene has 75% identity to human Mat2a and 73% identity to Mat1a, similar enough to be identified as a methionine adenosyltransferases, but not similar enough to be identified definitively as either one. It has a developmental expression profile, via RTPCR, similar to *mat1a*, but via in situ hybridization at 3 dpf we have not observed its expression being limited to the primitive liver (data not shown). At 1 dpf, its expression is enriched in the CNS (Figure 2).

Mthfd1 is a cytoplasmic trifunctional enzyme that interconverts methylene THF, methenyl THF, formyl THF, and formate. These folates serve as substrates for downstream reactions that synthesize thymidylate, methionine, and inosine. The protein is partitioned into an N-terminal bifunctional dehydrogenase and cyclohydrase domain (EC 1.5.1.5 and 3.5.4.9), and a C-terminal synthetase domain (EC 6.3.4.3). The mitochondrial protein, Mthfd1L retains a functional synthetase domain. However, while the N-terminal domain is present, mutations in critical residues results in the predicted loss of these enzymatic functions [17]. The mitochondrial dehydrogenase and cyclohydrase reactions are performed by the proteins Mthfd2 and Mthfd2L. Mthfd2 is only found in transformed mammalian cells and embryonic or nondifferentiated tissues [18] and is essential during embryonic development [19]. Mthfd2L has recently been shown to be the active mitochondrial enzyme in adult tissues [20]. Orthologs of all these human proteins are found in zebrafish. Two paralogs of Mthfd1 (NP_005947.3) are found in zebrafish: mthfd1a has 57% identity (74% similarity) to the human protein and is located on chromosome 20, mthfd1b has 77% identity (88% similarity) and is located on chromosome 17. Mthfd1b is the likely true ortholog of human Mthfd1 as it retains the conserved amino acids in the catalytic domains of this trifunctional enzyme. However, Mthfd1a has a predicted mitochondrial leader sequence (p = 0.9736, [21]) and has mutations in conserved catalytic residues in the synthetase domain. This transcript is dynamically regulated within the first 72 hpf. *Mthfd1b* is expressed throughout early development. At 24 hpf, both transcripts are enriched in the CNS, with mthfd1b also expressed in the YSL ([14], Figure 2). An ortholog of Mthfd1L (NP_056255.2) can be identified in zebrafish, on chromosome 20, with 81% identity (89% similarity) to the synthetase domain. While the zebrafish transcript is predicted to encode a mitochondrial leader sequence, the dehydrogenase/cyclohydrase domain has only 44% identity to the human protein. This transcript is not maternally expressed and is dynamically regulated throughout early development. Its expression is not spatially restricted at 24 hpf (Figure 2B). One ortholog of Mthfd2 (NP_006627.2) is found on chromosome 5 in zebrafish with 80% identity (92% similarity). An Mthfd2L ortholog is located on chromosome on 8, with 67% identity (82% similarity). Both of these transcripts are dynamically expressed throughout the first 72 hours of development, with expression at 24 hpf being enriched in the CNS (Figure 2).

Methylenetetrahydrofolate reductase (Mthfr, EC 1.5.1.20) catalyzes the irreversible reduction of the one carbon donor group on folate to 5-methyl-tetrahydrofolate. This form of folate is the carbon donor in the conversion of homocysteine to methionine. An ortholog of the human protein (NP_ 005948.3) is found in the zebrafish genome on chromosome 8 with 79% identity (89% similarity) (NP_001121727.1). This is also a maternally loaded transcript and is present throughout the early stages of development. At 24 hpf its expression is also enriched in the CNS and myotomes (Figure 2B).

Methionine synthase (Ms, EC 2.1.1.13), encoded by the gene *mtr*, is the enzyme that catalyzes the conversion of homocysteine to methionine, using 5-methyl-tetrahydrofolate as the methyl group donor and the vitamin cofactor cobalamin (vitamin B12). This enzyme sits at the intersection of the folate pathway and the methionine cycle. Zebrafish has one ortholog of this protein (NP_00245.2) on chromosome 12 with 77% identity (88% similarity). The transcript is maternally loaded and present throughout the first three days of embryonic development. At 24hpf its expression is enriched in the CNS ([14], Figure 2B).

Thymidylate synthetase (Tyms, EC 2.1.1.45) is an enzyme involved in a rate-limiting step of DNA synthesis: the methylation of dUMP to dTMP. This reaction uses a folate cofactor as the one carbon donor. An ortholog of the human protein (NP_001062.1) is found in the zebrafish genome on chromosome 7 with 82% identity (91% similarity). This is also a maternally loaded transcript and is present throughout the early stages of development. At 24 hpf its expression is also enriched in the CNS ([14], Figure 2B).

We also investigated the transport protein, Slc25a32 (MFTC), a mitochondrial membrane protein that transports folate into the mitochondrion [22]. Two paralogs of Slc25a32 (NP_110407.2) are found in zebrafish. *Slc25a32a* is located on chromosome 19 and has 66% identity (81% similarity) to the human protein. This transcript is also maternally expressed and is present throughout early development. At 24 hpf it is expression is not spatially restricted (Figure 2B). *Slc25a32b* is located on chromosome 16, has 70% identity (82% similarity) to the human protein, and is expressed throughout early embryonic development. Its expression is enriched in anterior neural tissues at 24 hpf (Figure 2B).

### Dhfr Inhibition

In order to investigate the role of the folate pathway in early zebrafish development, we inhibited Dhfr by using either the chemotherapeutic drug methotrexate or antisense morpholino oligonucleotides.

**Figure 3:**
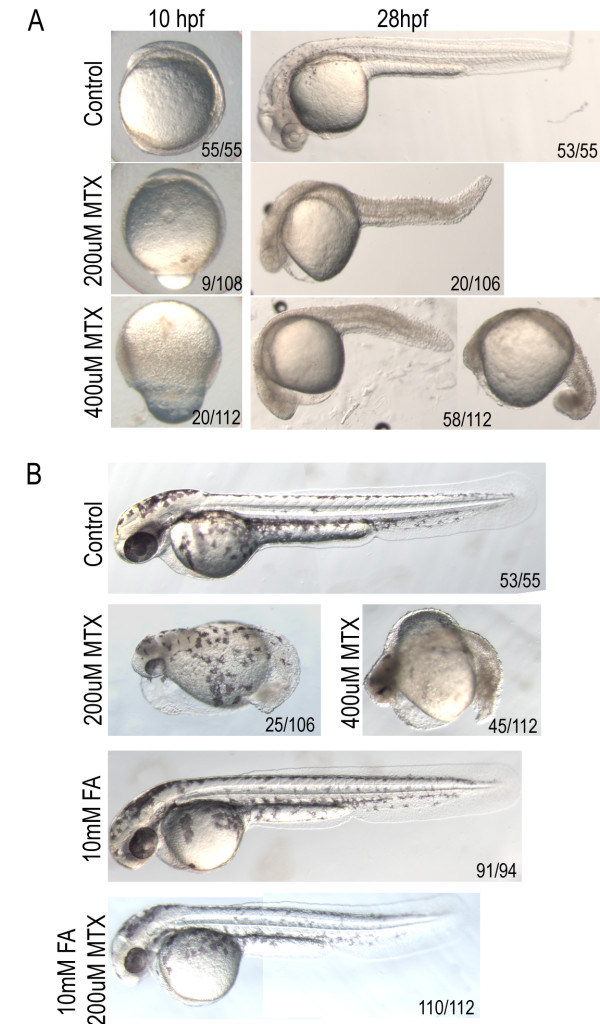
**Methotrexate treatment confers dose-dependent developmental delay.** A) Treatment with lower concentrations of MTX causes shortening of the anterior-posterior axis and heart defects, while higher doses result in embryonic lethality prior to somitogenesis. B) Defects evident at 2 dpf in embryos treated with 200 μM methotrexate are alleviated by treatment with 10 mM Folinic Acid (FA). Embryos in lateral view.

Methotrexate is a folate analog that acts by competitively inhibiting Dhfr [23]. Zebrafish embryos were treated with varying concentrations of methotrexate, within 30 minutes of fertilization. At the highest concentration of methotrexate tested, 600 μM, most embryos died prior to 1 dpf. The few surviving embryos were morphologically abnormal, arresting between epiboly and early somitogenesis. As the concentration of methotrexate decreased, the severity of the phenotype diminished, but even the lowest concentration of methotrexate tested, 100 μM, resulted in embryonic lethality by 5 dpf. These fish were characterized by ventral edema, dorsal curvature, shortened A/P axis, and defective heart morphology. Embryos treated with 200 μM methotrexate had a mild delay at tailbud stage. By 1 dpf, these embryos displayed necrotic heads and tails with dorsal curvature and a shortened A/P axis (Figure 3A). Embryos developed ventral edema and heart defects that ultimately resulted in embryonic lethality by day 5. The majority of embryos treated with 400 μM methotrexate resulted in embryonic lethality prior to somitogenesis (Figure 3A). A minority of embryos developed past early somitogenesis but had severely shortened A/P axis with necrosis throughout the embryo and cardiac defects.

Folinic acid, a folate derivative that can enter the folate metabolic pathway without the enzymatic function of Dhfr, is used during chemotherapy to ameliorate the toxic effects of methotrexate [24]. Embryos pre-treated with folinic acid in addition to 200 μM methotrexate showed fewer severe defects at 2 dpf: less edema, fewer embryos with dorsal curvature and a decrease in cardiac defects (Figure 3B). However, this treatment was unable to completely rescue methotrexate treatment as the embryos died by 5 dpf.

**Figure 4:**
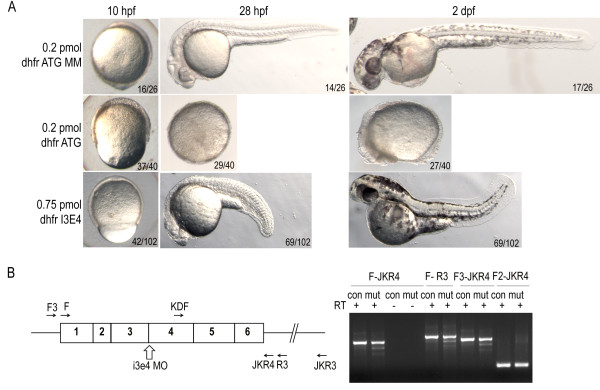
***dhfr*morpholino injection phenocopies methotrexate treatment defects.** A) Epiboly in *dhfr* ATG morphants is significantly delayed at 10 hpf as compared to controls. By 28 hpf severe *dhfr* ATG morphants are developmentally arrested with a failure to complete epiboly. Intron-exon splice morphants complete epiboly but exhibit shortened anterior-posterior axis and heart defects. Embryos in lateral view. B) Diagram of *dhfr* mRNA, i3e4 splice blocking morpholino, and primers used for RT-PCR (sequences supplied in Additional file 2: Table S2). cDNA generated from embryos at 6 hpf demonstrate splicing defects in *dhfr* transcripts in embryos treated with morpholino (mut). Control injected embryos (con) have only expected band sizes.

We confirmed the specificity of the observed defects being due to abrogation of the folate pathway by using morpholinos against *dhfr*. The same spectrum of defects was observed when embryos were treated with either translation or splice blocking morpholinos. Microinjection of 0.2 pmol of translation blocking morpholino [13] resulted in over 50% of embryos displaying the severe defect of developmental arrest after tailbud stage (Figure 4A). Less than 10% of embryos treated with 0.75 pmol of splice blocking morpholino, designed to bind the intron 3- exon 4 splicing boundary, exhibited this severe early phenotype (Figure 4A). RT-PCR analysis of *dhfr* in treated embryos showed laddering bands characteristic of splicing defects (Figure 4B) [25]. Sequencing results from these aberrant bands demonstrated that these bands were the result of skipping either exons 3 and 4 or just exon 4 and resulted in a pre-mature termination codon (data not shown). With this treatment, most embryos exhibited shortened anterior-posterior axis, ventral edema, dorsal curvature, cardiac defects, and a noticeable decrease in their touch response, culminating in embryonic lethality. In contrast, injection of 0.2 pmol of the 5 bp mismatch (MM) translation blocking morpholino control resulted in 90% viability at 5 dpf. Co-injection with a morpholino targeting p53, which blocks nonspecific morpholino induced apoptosis, resulted in similar phenotypes as the *dhfr* ATG morpholino alone (data not shown).

### Differentiation and tissue specification

**Figure 5:**
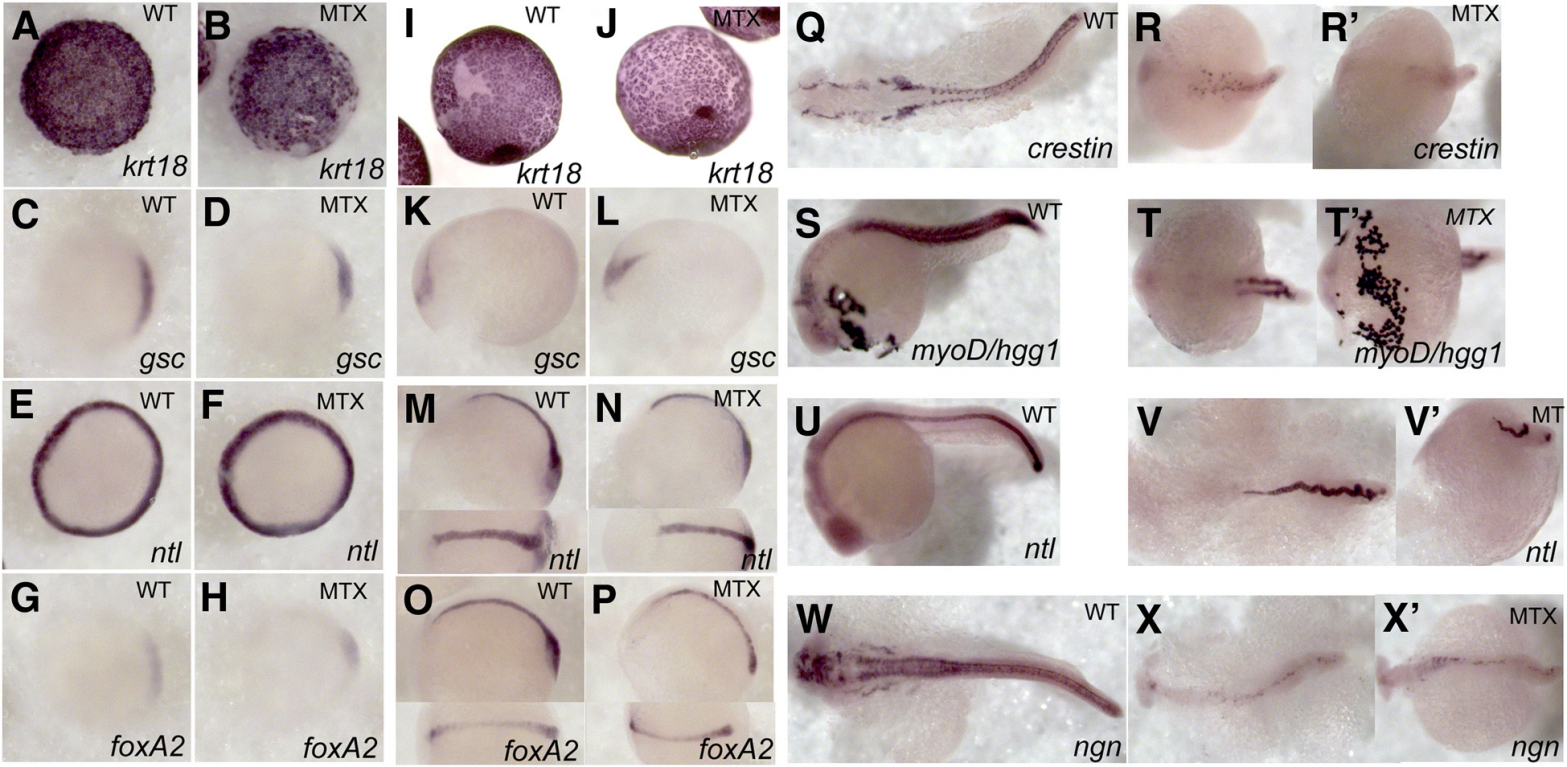
**Methotrexate–treated embryos maintain expression of appropriate developmental markers.** Expression patterns were visualized via whole-mount *in situ* hybridization in embryos treated with 400 μM MTX and fixed at shield stage (A-H), tailbud (I-P), and 24 hpf (Q-W’). While MTX-treated embryos at 24hpf are clearly delayed with respect to normal development, they do express appropriate developmental markers: *krt18* –enveloping layer, *gsc* – involuting cells and neural plate, *ntl* – blastomere margin, notochord and dorsal organizer, *foxA2* – involuting cells and neural plate, *crestin* – neural crest, *myoD* – paraxial mesoderm, *hgg*- hatching gland (anterior most structure), *ngn* – CNS. (A-J) Animal view, dorsal to the right. (K-P) Lateral view with dorsal view inset. (Q, R, R’, T, V, W, X, X’) Dorsal view. (S, U, V’) Lateral view. (T’) ventral view.

The developmental defects observed in fish with disruptions in one carbon metabolism could be due to failure of differentiation. To test this we performed *in situ* analysis of markers of primarily neuro-ectodermal tissues. This experiment demonstrated that while phenotypically abnormal, embryos treated with 400 μM methotrexate were able to specify developmentally appropriate tissues (Figure 5). The enveloping layer (*krt18*) develops and migrates, despite delays and defects in blastopore closure. Treated embryos form a dorsal shield with involuting cells (as indicated by *gsc* and *foxa2 in situ* hybridization), which gives rise to neural plate and neural tube structures, respectively, by bud stage. *ntl* indicates margin cells during shield stage and notochord at bud stage. By 24 hpf, *ntl* staining indicates an abnormal shortened wavy notochord. This observation is supported by somite staining with *myoD*. *foxA2* and *crestin*, a marker of neural crest, also showed an abnormal staining pattern, but demonstrates that tissues are specified. Finally, the hatching gland, *hgg1*, indicates the anterior most structure of the zebrafish is also specified. The endothelial marker *kdrl* was appropriately specified, as were the anterior neural markers *wnt1*, *sox3*, and *pax6a* (data not shown). While tissues queried were all appropriately specified, the notochord is abnormal and could indicate a defect in the overlying neural tube.

### Cell cycle aspects of folate pathway perturbations

Embryos treated with methotrexate are phenotypically abnormal but tissues are normally specified. We hypothesized the defect is in the number of cells in the embryo, due to defects and delays in the cell cycle in response to reduced folate substrate for the enzyme thymidylate synthetase.

**Figure 6:**
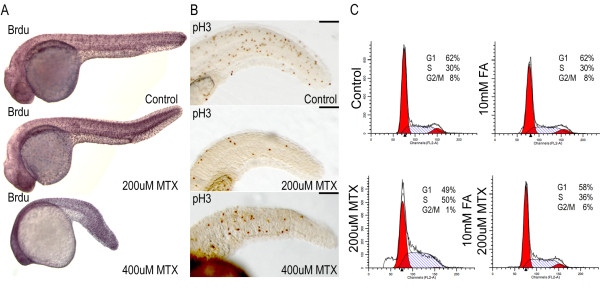
**Abnormal cell cycle properties of methotrexate treated embryos A) After 24 hours of 200 μM or 400 μM methotrexate exposure, embryos are capable of incorporating BrdU via DNA synthesis.** B) Immunohistochemistry for the mitotic marker phospho-histone H3 shows a decreased number of mitotic cells in methotrexate treated embryos. C) However, FACS analysis clearly indicates methotrexate treated embryos do not display WT cell cycling profiles, with a probable S-phase accumulation. Co-treatment with folinic acid and methotrexate restores cell cycle profiles. Lateral views of 24 hpf embryos. Scale bar: 100 μm.

Methotrexate treated embryos were assessed in their ability to incorporate the thymidine analog, BrdU, into their DNA. Embryos treated with methotrexate readily incorporated BrdU into their DNA, demonstrating competence in S-phase (Figure 6A). This assay is not sensitive enough to detect an increase in the number of cells in S-phase. In order to assess cells in M-phase, we performed immunocytochemistry using an antibody to phospho-histone H3, a mitosis specific marker. Embryos treated with methotrexate showed a decreased number of phospho-histone H3 positive cells (Figure 6B). These data were supported by DNA content analysis via flow cytometry. Embryos treated with 200 μM methotrexate for 24 hours showed an increased proportion of cells in S-phase with a concomitant decrease in cells in M-phase (Figure 6C). This defect was not observed in embryos pre-treated with folinic acid. Embryos treated with 400 μM methotrexate demonstrated a severe skewing of cells to the 2 N and sub-2 N DNA content. This sub-2 N DNA peak suggests an increase in apoptotic cells. To test this possibility we performed TUNEL assays on treated and untreated embryos. Embryos treated with methotrexate demonstrated an increase in apoptotic cells (data not shown). Methotrexate treatment causes S-phase delay, a decrease in the number of mitotic cells, and an increase in apoptosis, resulting in an overall decrease in the number of cells within the developing embryo.

## Discussion

Our aim was to identify and characterize components of the folate pathway and to interrogate its role in early zebrafish development. Twelve genes involved in folate metabolism were identified by BLAST searches, cloned, and their expression profiles studied by RT-PCR and RNA *in situ* hybridization. We also assessed the impact of inhibiting the folate pathway in the developing zebrafish by treatment with the folate antagonist, methotrexate, and by microinjection of antisense morpholino oligonucleotides designed to disrupt Dhfr expression. Inhibition of the folate pathway caused perturbations in the cell cycle, which resulted in embryonic lethality. While it is not surprising that this pathway is essential for development, the mechanism through which this inhibition acts had not been explored. We propose the primary defect in these fish is the lack of folate substrates for Tyms, a rate-limiting step in DNA synthesis, which results in the lack of dTTP pools for DNA synthesis. However, it is also possibile that other methylation reactions, requiring s-adenosylmethionine (a downstream product of folate one- carbon pathway), also play a role in the defects we observed.

We set out to identify and clone 12 orthologs of folate genes in humans. We identified 16 orthologs and paralogs, presumably due to the partial genome duplication that occurred in teleosts [26]. The presence of paralogs can complicate studies that try to establish gene function by looking at single gene knock-out (down) phenotypes. One set of duplicated genes we identified was the *mat*s: *mat1a* and *mat2a*. *Mat1a* encodes the liver specific isoform of the enzyme. *Mat2a* encodes the globally expressed isoform. Zebrafish has four *mat* genes. One is clearly *mat1a*, with 77% identity to the human protein and liver expression at 3 dpf (data not shown). Two genes, *mat2aa* and *mat2ab*, appear to be paralogs of human *mat2a*, with 80% identity to the human protein and without liver restricted expression. The fourth gene, *mat2al*, has a temporal expression pattern similar to *mat1a*, but without the liver restricted expression, similar to the *mat2a*s, and relatively low (75% and 73%) identity to the human Mat1a and Mat2a proteins, respectively. This highlights one of the challenges of transferring pathways between species.

Queried members of the folate pathway are generally maternally loaded and ubiquitously expressed (with enrichment in anterior neural tissues), consistent with the idea that they are house-keeping genes involved in an essential cellular process. Inhibition of the pathway with either the folate antagonist methotrexate or by using morpholinos targeting *dhfr* results in early embryonic lethality. Further study of methotrexate treated embryos indicates defects in the cell cycle, specifically S-phase accumulation and increased apoptosis. By visual morphological criteria and *in situ* analysis embryos have a normal developmental program through bud stage. Between 12 hpf and 24 hpf development defects arise, as >80% of embryos treated with 400 μM methotrexate die overnight and the surviving embryos are anatomically abnormal. Neural cells condense to form the neural plate around the 2 somites stage (11 hpf) [10]. Most embryos treated with methotrexate do not survive treatment to be queried specifically for neural tube defects. Despite decreased cell numbers due to S-phase delay and concomitant decreased mitosis, the developmental program proceeds and appropriate tissues are specified, although the anterior-posterior axis is severely shortened and the notochord undulates. Convergent/extension defects, resulting in embryos with a shortened A-P axis, are observed in zebrafish expressing rare variants of VANGL1 that are associated with neural tube defects in humans [27].

One limitation of using zebrafish as a model system for understanding genetic and environmental etiologies of neural tube defects is that the mechanics of zebrafish neurulation differs from that of human and mouse. Zebrafish form a neural plate, which then progresses through a neural keel stage, to a solid neural rod which then cavitates to a neural tube [10]. Zebrafish do not form a neural tube via the bending of the neural plate and fusing of opposing sides. In humans and mice, the failure of these tissues to fuse presents as a neural tube defect, whether in its lethal form of anencephaly or the milder state of spina bifida. However, many of the molecular and genetic aspects of neural tube formation are genetically conserved between humans and fish [28].

The essential requirement for the folate pathway has been documented in other model organisms ranging from yeast to mice. Of the genes we studied, only dfr1 (*dhfr*) and cdc21 (*tyms*) are essential in budding yeast. *Mtr −/−* mice are embryonic lethal prior to day E9.5 [7]. *Mthfd1* −/− mice also are embryonic lethal [29] as are *Mthfd2 −/−* mice, by day E13.5, and can be distinguished by their smaller size and pale livers [19]. In contrast *Mthfr* −/− knock out mice are viable, although with reduced viability, decreased body size, and cerebellar pathology [30]. A small number of reports have investigated folate pathway genes and mutants in zebrafish. Sun *et al.*[13] reported cardiac defects in zebrafish *dhfr* morphants. Similar cardiac defects were also reported for zebrafish embryos treated with methotrexate, as well as decreased expression of the cardiac markers (*hand2, mef2a,* and *mef2b*) [31]. Neither study reports the early severe defects we observed nor do they interrogate cell cycle properties of affected embryos. Zebrafish *tyms* mutants were identified in screens looking for genes essential for embryonic [32] or defective in retinal development [33]. Another zebrafish mutant with ocular defects has been identified as *gart*, one of two enzymes involved in purine metabolism that uses folate as a cofactor [34]. Our identification and characterization of major components of the folate pathway in zebrafish increases the utility of this model organism in studying the role of folate in normal development and disease.

## Conclusion

We provide the first in depth characterization of the folate pathway in zebrafish, by reporting the expression patterns of 16 genes, of which we name 6 genes in ZFIN and provide additional sequence information for 3 genes. We expand upon earlier studies that implicated Dhfr in cardiac development by identifying an earlier defect in zebrafish. Folate starved embryos start to show defects prior to segmentation. We propose this is due to defects in S phase, caused by decreased pools of thymidylate and consequently dTTP, which in turn leads a lengthened cell cycle. As each round of division takes longer, fewer total cells are available in which to form an embryo. We also observed an increase in apoptosis, which we propose to be a consequence of and in response to extended delays in S-phase. Despite the decreased number of cells and decreased size of the entire organism, mutant embryos are able to appropriately specify the neuro-ectodermal and mesodermal tissues that were queried. The typical consequence of folate deficiency is megaloblastic anemia, an enlargement of red blood cells, due to the failure of cellular division of erythrocyte precursors. Our data support the idea that in rapidly dividing hematopoietic or embryonic cells, DNA synthesis is susceptible before purine synthesis and other cellular methylation reactions. Low folate status and hyperhomocysteinemia have been implicated as potentially reversible risk factors for cognitive decline and cardiovascular disease. Our work establishes zebrafish as a model system for studying the folate pathway and demonstrates that zebrafish can serve as a model organism for studying the role of folate in development in order to understand the genetic and environmental etiology underlying some neural tube and congenital heart defects.

## Methods

### Zebrafish care

The zebrafish, TAB-5 strain (ZIRC, Eugene, OR), were bred and maintained at 28.5°C as described [35]. Animals were staged by hours post-fertilization (hpf) and on morphological criteria. Embryos were treated with Methotrexate USP (GeneraMedix, Liberty Corner, NJ) and/or Leucovorin USP (folinic acid) (Bedford Laboratories, Bedford, OH) within 30 minutes of fertilization in 1X E3. We treated embryos with 5 orders of magnitude more than the minimal chemotherapeutic dose of methotrexate. We used ^14^ C labeled methotrexate to measure intra-embryonic uptake of the drug. These data suggest that only small amounts of drug permeate the chorion into the embryo (data not shown). Embryos were injected with up to 4 nL of diluted morpholino (Genetools LLC, Philomath, OR) (as determined by drop size in oil) prior to 8-cell stage. Unfertilized embryos were removed between 4–6 hpf and media was changed at 2 dpf. Experimental protocols were reviewed and approved by the NHGRI Animal Use and Care Committee. Morpholino sequences: tp53 GCGCCATTGCTTTGCAAGAATTG [36], dhfr ATG 5’ ACGGTCTCGCCTTCTTCCCGCCAAG 3’ [13], dhfr.i3e4 5’ TGTCCTGTGGAGTGACAGAAAATAA 3’, dhfr.MM 5’ ACcGTCTCGgCTTgTTCgCGCCgAAG 3’ (mismatches in lower case).

### Cloning

Orthologs were identified by TBLASTN of the human protein sequence to the zebrafish genome (Ensembl v7) and/or cDNAs. Primers to predicted transcripts were designed to amplify the 5’UTR and CDS. RNA was isolated from 24 hpf embryos using RNA STAT-60 (Amsbio, Abingdon, UK) and transcribed to cDNA (Superscript III, Invitrogen, Carlsbad, CA). Transcripts were amplified (Hotstar Taq, Qiagen, Valencia, CA), cloned into pCRII (Invitrogen, Carlsbad, CA), and sequenced. **GenBank accession numbers**: JN848834 (*mat2aa*), JN848835 (*mat2ab*), JN848836 (*mthfr*).

### RT-PCR

RNA was isolated from staged embryos (4 cell through 72 hpf) using RNA STAT-60 (Amsbio, Abingdon, UK) or TRI reagent LS (Sigma-Aldrich, St. Louis, MO). 1 μg of total RNA was transcribed into cDNA (Superscript III SuperMix, Invitrogen, Carlsbad, CA). Primers for RT-PCR were designed to yield 400–600 bp products, biased to the 3’ end of the transcript, and spanned at least one intron - exon boundary ( Additional file 1: Table S1). Multiple overlapping sets of primers were designed to the *dhfr* transcript to query the efficacy of morpholino induced knock-down ( Additional file 2: Table S2). Aberrant splice products were TOPO-TA cloned (Invitrogen, Carlsbad, CA) and sequenced.

### *In situ* hybridization

In situ probes were designed to be ~500 bp and biased to the 3’UTR. Hybridizations were performed essentially as described [37].

### FACS

Embryos were treated with 400 μM methotrexate for 24 hours. 20–40 embryos were processed in duplicate. After treatment, embryos were briefly rinsed in calcium-free Ringers solution (116 mM NaCl, 2.9 mM KCl, 5 mM HEPES pH 7.2), pestled 20 times in calcium-free Ringers, and then passed through 40 μM filter (BD Falcon 352340), rinsed once and resuspended in DNA staining buffer (100 mM Tris pH 7.5, 154 mM NaCl, 1 mM CaCl, 0.5 mM MgCl, 0.2% BSA, 0.1% Nonidet P-40, 250ug/mL Rnase, 20ug/mL propidium iodide) [38]. FACS analysis was performed on FACSCalibur (BD Biosciences) and data analyzed in FlowJo (Ashland, OR).

### BrdU and phospho-histone H3 Assays

Phosphohistone-H3 immunochemistry was performed as described [39]. BrdU incorporation was assayed essentially as described [40] with the following modifications: fixed embryos were permeabilized with methanol and treated with 4 M HCl for 20 minutes. Primary mouse anit-BrdU antibody (clone II5B, Santa Cruz) was used at a 1:250 dilution and secondary AP-conjugated donkey anti-mouse antibody was diluted 1:500. Alkaline phosphatase was developed with NBT/BCIP solution (Roche, Basel, Switzerland).

## Abbreviations

FOCM, Folate-one carbon metabolism; NTD, Neural tube defects; MTX, Methotrexate; SAM, s-adenosyl methionine; FA, Folinic acid; hpf, hours post-fertilization; dpf, days post-fertilization; A/P, Anterior-posterior.

## Authors’ contributions

MSL designed and performed all the experiments. SMB, LCB contributed to experimental design and planning. JRB, DJB, ELS, ETS., RRP aided in gene identification and cloning. JRB performed morpholino injections. RRP and JRB did preliminary methotrexate experiments. ETS performed *in situ* hybridizations. MSL and LCB drafted the manuscript. All authors read and approved the final manuscript.

## Supplementary Material

Additional file 1 Table S1. Primers for *in situ* and RTPCR of folate pathway genes. Click here for file

Additional file 2 Table S2. Primers sequences for *dhfr* RTPCR. Click here for file
